# The biological response to nanometre-sized polymer particles

**DOI:** 10.1016/j.actbio.2015.05.016

**Published:** 2015-09-01

**Authors:** Aiqin Liu, Laura Richards, Catherine L. Bladen, Eileen Ingham, John Fisher, Joanne L. Tipper

**Affiliations:** Institute of Medical and Biological Engineering, University of Leeds, Leeds LS2 9JT, UK

**Keywords:** Nanoparticle, Polyethylene, Bioactivity, Osteolysis, Joint replacement

## Abstract

Recently, nanometre-sized UHMWPE particles generated from hip and knee replacements have been identified *in vitro* and *in vivo.* UHMWPE particles in the 0.1–1.0 μm size range have been shown to be more biologically active than larger particles, provoking an inflammatory response implicated in late aseptic loosening of total joint replacements. The biological activity of nanometre-sized particles has not previously been studied. The biological response to clinically-relevant UHMWPE wear particles including nanometre-sized and micrometre-sized, along with polystyrene particles (FluoSpheres 20 nm, 60 nm, 200 nm and 1.0 μm), and nanometre-sized model polyethylene particles (Ceridust 3615®), was determined in terms of osteolytic cytokine release from primary human peripheral blood mononuclear cells (PBMNCs). Nanometre-sized UHMWPE wear particles, nanometre-sized Ceridust 3615® and 20 nm FluoSpheres had no significant effect on TNF-α, IL-1β, IL-6 and IL-8 release from PBMNCs at a concentration of 100 μm^3^ particles per cell after 12 and 24 h. The micrometre-size UHMWPE wear particles (0.1–1.0 μm) and 60 nm, 200 nm and 1.0 μm FluoSpheres caused significantly elevated osteolytic cytokine release from PBMNCs. These results indicated that particles below circa 50 nm fail to activate PBMNCs and that particle size, composition and morphology played a crucial role in cytokine release by particle stimulated macrophages.

## Graphical abstract

**Figure:**
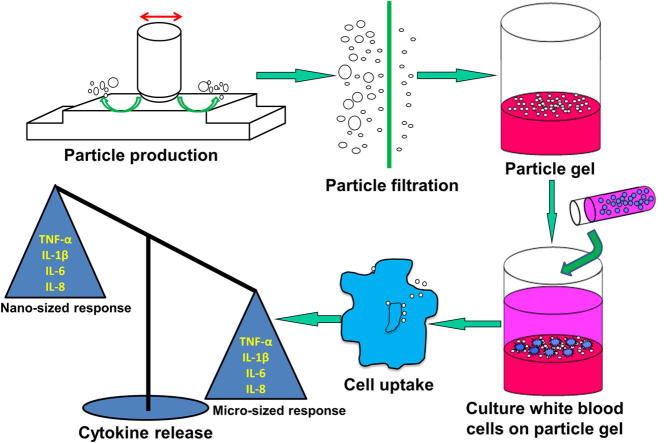

## 1 Introduction

UHMWPE wear particles have been implicated in late aseptic loosening of conventional total hip replacements (THR) [1,2]. As these particles are generated and released into the tissue surrounding the implant, macrophages attempt to digest the foreign material. Polyethylene is, however, inert and it cannot be broken down. The macrophages become activated and release pro-inflammatory mediators such as tumour necrosis factor-α (TNF-α) and interleukins, IL-1β, IL-6 and IL-8. In a complex sequence of events osteoclasts are activated to resorb the bone at the bone-implant interface and an imbalance in normal bone metabolism results in painful loosening of the prosthesis [3–5].

Alternative bearing materials such as metals and ceramics with lower wear rates were introduced to solve the problems associated with particle-induced osteolysis [6]. However, the high failure rate of metal on metal bearings as well as serious adverse tissue reactions [7–14] and the high cost of ceramic bearings have led to UHMWPE bearings remaining dominant in THR. Currently, the most common bearing surface material in THR is highly crosslinked UHMWPE, which has been successfully used in the clinic for more than a decade.

The osteolytic potential of the UHMWPE wear particles is dependent on particle size and volume. It has been reported that UHMWPE particles in the size range of 0.1–1.0 μm are more biologically active than larger particles in terms of osteolytic cytokine release [15,16]. Recently, nanometre-sized UHMWPE particles have been identified both in *in vitro* wear test lubricants and in tissues retrieved at revision surgery [17–20]. Most importantly, highly crosslinked UHMWPEs have been shown to produce large numbers of nanometre-sized wear particles and generate higher volumes of nanometre-sized wear particles compared to conventional non-crosslinked UHMWPE [21,22]. Previous studies that have assessed the biological activity of UHMWPE particles *in vitro* have not considered particles in the nanometre-size range [15,16,23,24]. Therefore, it is essential to determine the relative contribution of particles less than 100 nm in size to the inflammatory process of osteolysis in total hip replacements.

The aims of this study were to determine the capacity of nanometre-sized polymer particles to activate human peripheral blood derived mononuclear phagocytes (PBMNC) to produce osteolytic cytokines *in vitro*. Particles investigated included commercially available model particles (FluoSpheres and Ceridust 3615®) and clinically-relevant UHMWPE particles that were generated by multidirectional articulation. A large volume of endotoxin-free clinically-relevant nanometre-sized UHMWPE particles was generated using RPMI 1640 medium lubricant in multi-directional pin-on-plate wear tests. Particles were cultured with PBMNCs isolated from healthy volunteers and cell viability and cytokine release were measured.

## 2 Materials and methods

### 2.1 Preparation of polymer particles

#### 2.1.1 Generation of micrometre-sized and nanometre-sized UHMWPE wear particles

UHMWPE wear particles were generated from GUR 1020 GVF UHMWPE pins (gamma irradiated at a dose of 2.5–4 MRad in a vacuum; bar stock supplied by DePuy International Ltd., UK) against medical grade high carbon (>0.2%, w/w) cobalt chromium alloy plates (School of Mechanical Engineering, University of Leeds, UK) with a surface roughness (Ra) of 0.07–0.08 μm using a six station multi-directional pin-on-plate wear simulator. RPMI 1640 medium was used as the lubricant (Bio-Whittaker, Lonza, Belgium) according to established methods detailed in our previous study [25]. Post-test lubricants containing UHMWPE wear particles were collected. Micrometre-sized UHMWPE wear particles (Micro-wear-0.1–1.0 μm) were isolated from the sonicated wear test lubricants by filtration through 10 μm, 1 μm, 0.6 μm and finally onto a preweighed 0.1 μm polycarbonate membrane filter (Whatman, Kent, UK). Larger micrometre-sized UHMWPE wear particles (Micro-wear-1.0–10 μm) were isolated from wear test lubricants by filtration through a 10 μm filter and then onto to a preweighed 1 μm filter. The 0.1 μm and 1 μm filters containing particles were dried under infrared lamps for 4 h and weighed 5 times using a microbalance with an accuracy of 1 × 10^−6^ g in an environmentally controlled room. The mass of micrometre-sized particles on the 0.1 μm filter and the 1 μm filter was calculated. A filter sequence of 10 μm, 1 μm, 0.1 μm, 0.1 μm and 0.015 μm pore sizes was used to isolate nanometre-sized UHMWPE wear particles (<100 nm) on the 0.015 μm filters. The mass of nanometre-sized UHMWPE wear particles on the 0.015 μm filter was determined as above.

#### 2.1.2 Commercially available polystyrene FluoSpheres®

FluoSpheres® (FS, Invitrogen Life Technologies Ltd., Paisley, UK) were purchased in 20 nm, 40 nm, 200 nm and 1.0 μm sizes and stored at 4 °C with protection from light.

#### 2.1.3 Commercially available polyethylene resin, Ceridust 3615®

Ceridust 3615®-a low molecular weight polyethylene resin (Hoechst, Germany) was dispersed in a solution consisting of RPMI 1640 medium plus 0.08% (v/v) 7X-PF detergent (ICN Biomedicals Inc., USA). After sonication for 40 min, the suspension was filtered through a filter sequence of 10 μm, 1 μm, 0.1 μm, 0.1 μm and finally onto a preweighed 0.015 μm pore sizes. The mass of particles collected on the 0.015 μm filter was determined.

### 2.2 Characterisation of polymer particles

The polymer particles were characterised using images captured by high resolution field emission gun-scanning electron microscopy (FEG-SEM, LEO Electron Microscopy Ltd., Cambridge, UK). Each type of fractionated polymer particle was suspended in RPMI 1640 medium with 20 mM HEPES (N-(2-hydroxyethyl) piperazine-N′-(2-ethanulfonic acid) (Bio-Whittaker, Lonza, Belgium) and 100 U ml^−1^ penicillin/streptomycin (Bio-Whittaker, Lonza, Belgium). A volume of 24 μl of FluoSpheres, 100 μl of fractionated nanometre-sized Ceridust and 100 μl of each of the UHMWPE wear particle suspensions was resuspended in 70% (v/v) ethanol (VWR International Poole, UK) and filtered through a 0.015 μm filter. Sections of each filter were mounted on aluminium stubs using double sided carbon pads and coated with 3.0 nm platinum/palladium using a sputter coater (Agar Scientific Ltd., UK). A minimum of 100 particles were analysed for each sample using a wide range of magnifications (×1000–200,000). The particle distributions were determined using Image Pro Plus software (Media Cybernetics, Maryland, USA) by measuring the size (length) and area of particles on the filters.

### 2.3 Endotoxin testing of polymer particles using the LAL assay

The Pyrochrome Limulus Amebocyte Lysate (LAL) kinetic-QCL endotoxin assay (Associates of Cape Cod Incorporated, USA) was used to determine the presence of endotoxin in all particle stocks. Particle samples were diluted 1:1000 using LAL reagent water. A series of four endotoxin standards (5 EU ml^−1^, 0.5 EU ml^−1^, 0.05 EU ml^−1^ and 0.005 EU ml^−1^) were generated using Control Standard Endotoxin (CSE, 10 ng/vial, which was reconstituted to give a concentration of 100 EU ml^−1^). A 96-well plate loaded with 1:1 ratio of samples and pyrochrome reconstitution reagent was placed in an absorbance micro plate reader (Bio-Tek, USA), shaken for 10 s and the assay was carried out at 37 °C for 1.5 h. A measurement filter of 405 nm was used. The test system was set up using KC4 software. The concentration of endotoxin present in the particle suspensions was calculated from reaction time using a standard curve, where the rate of colour change was directly proportional to the amount of endotoxin present.

### 2.4 Culture of human peripheral blood mononuclear cells (PBMNCs) with polymer particles

Heparinised blood (30 ml) was obtained by venepuncture of 9 healthy volunteer donors (aged 20–55 years, 6 females and 3 males). All blood was collected in accordance with the Faculty of Biological Sciences Ethics Committee approval (R1307 and BIOSCI 10-018) and informed consent was obtained from the donor prior to venepuncture. The mononuclear cell fraction (PBMNC) was isolated by density gradient centrifugation on Lymphoprep (Axis-Shield PoC AS, Oslo, Norway). The PBMNCs were collected and suspended in a known volume of cell culture medium (RPMI 1640 medium with 10% (v/v) foetal bovine serum (Bio-Whittaker, Lonza, Belgium), 2 mM l-glutamine (Bio-Whittaker, Lonza, Belgium) and 100 U ml^−1^ penicillin/streptomycin (Bio-Whittaker, Lonza, Belgium). The number of phagocytic cells was determined using a latex bead ingestion assay [26].

Particles were cultured with PBMNCs using an agarose gel technique as described previously [27]. Cells were seeded at 1.125 × 105 cells per well in 48 well plates containing particles in low melting point agarose (Invitrogen Life Technologies Ltd., Paisley, UK) at a concentration (particle volume (μm^3^): cell number ratio) of 100:1 (*n* = 4). A volume of 1.125 × 107 μm^3^ particles was required for each well to give a concentration of (particle volume (μm^3^): cell number ratio) of 100:1. The density of polyethylene wear particles was assumed to be 1 × 10^−6^ μg μm^−3^. So the required mass of wear particles per well was calculated by the equation below:

$$\begin{array}{c} \text{Weight of particles}=\text{Volume of particles}\times \text{Density}\\ =1.125\times 10^{7}\;\mu {\text{m}}^{3}\times 1\times 10^{-6}\;\mu \text{g}\;\mu {\text{m}}^{-3}\\ =11.25\;\mu \text{g} \end{array}$$

Cells cultured without particles (*n* = 4) were used as a negative control and cells treated with 200 ng ml^−1^ of lipopolysaccharides (LPS) (*n* = 4) (Sigma–Aldrich, St. Louis, MO, USA) were used as a positive control. Plates were seeded and then incubated in an atmosphere of 5% (v/v) CO_2_ in air and 100% humidity at 37 °C for 12 and 24 h. Culture supernatants were harvested and stored at −20 °C in 96 well polypropylene plates for a maximum of one month for cytokine measurement. Cell viability was determined by ATP Lite assay (PerkinElmer, USA). TNF-α and interleukin (IL-1β, IL-6 and IL-8) release was measured by enzyme linked immunosorbent assay (ELISA, IDS, Michigan, USA) at 12 and 24 h.

In order to visualise the cellular uptake of UHMWPE wear particles within agarose gels using confocal laser scanning microscopy, UHMWPE wear particles were labelled with sodium fluorescein, according to methods described in a previous study [28]. The nanometre-sized (<100 nm) and 0.1–1.0 micrometre-sized UHMWPE wear particles were stained using fluorescein solution (1 mg ml^−1^; Fisher Scientific Ltd., UK) at a concentration of 1:10 at 4 °C. PBMNCs were cultured in custom made wells on microscope slides (Thermo Scientific, Menzel-Glaser, UK) with green fluorescently labelled nanometre-sized (<100 nm) and 0.1–1.0 micrometre-sized UHMWPE wear particles at a concentration of 100 μm^3^ per cell for 48 h using the agarose gel method. The nuclei of cells were stained with Hoechst 33342 (Life Technologies, UK) at a concentration of 1.0 μM. Cells were then imaged using a confocal laser scanning upright Zeiss LSM510 microscope (Carl Zeiss Ltd., UK).

### 2.5 Statistical analysis

Data are shown as mean ± 95% confidence interval and were analysed by a one-way ANOVA. Differences between the treatment groups and the negative control were determined by calculating the minimum significant difference (MSD) (*p* < 0.05) by the Tukey-method.

## 3 Results

### 3.1 Characterisation of polymer particles

**Fig. 1:**
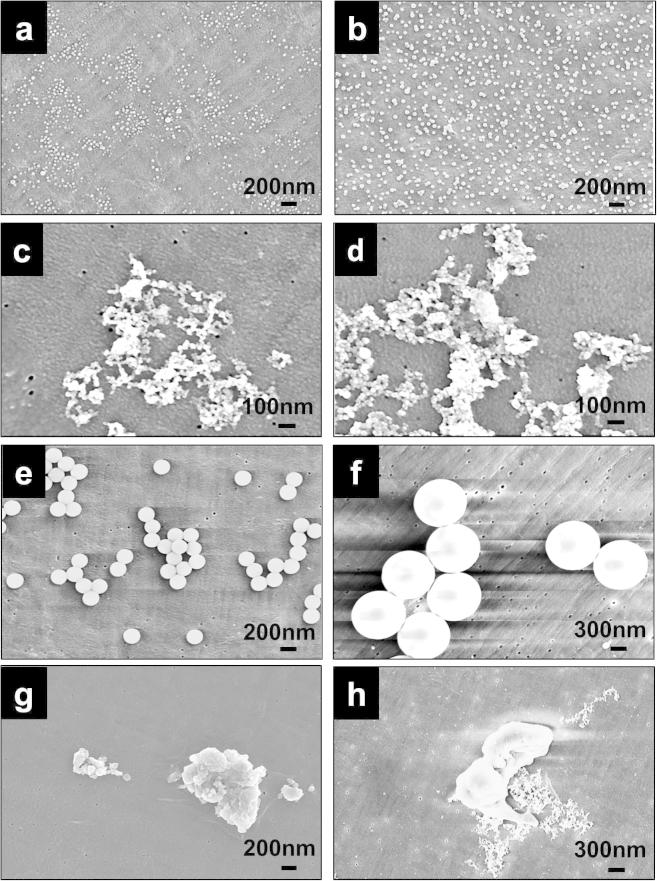
FEG-SEM images of FluoSpheres (FS), Ceridust 3615® and UHMWPE wear particles: (a) 20 nm FS, magnification of 90,000×; (b) 40 nm FS, magnification of 90,000×; (c) nanometre-sized Ceridust 3615® particles, magnification of 200,000×; (d) nanometre-sized UHMWPE wear particles, magnification of 200,000×; (e) 200 nm FS, magnification of 90,000×; (f) 1 μm FS, magnification of 60,000×; (g) UHMWPE wear particles (Micro-wear-0.1–1.0 μm), magnification of 90,000×); (h) UHMWPE wear particles (Micro-wear-1.0–10 μm), magnification of 65,000×.

The morphologies of nanometre-sized polymer particles are shown in Fig. 1a–d. The 20 nm and 40 nm FS on the 0.015 μm filter had regular round morphologies (Fig. 1a and b). Agglomerated granular nanometre-sized Ceridust 3615® particles were observed clearly at a magnification of 200,000× (Fig. 1c). Agglomerated nanometre-sized granular UHMWPE wear particles with a size range of less than 50 nm were abundant on the 0.015 μm filter (Fig. 1d).

The morphologies of micrometre-sized polymer particles are shown in Fig. 1e–h. The 200 nm FS and 1 μm FS on the 0.015 μm filters had regular round morphologies (Fig. 1e and f). The UHMWPE wear particles (Micro-wear-0.1–1.0 μm) isolated using a filter sequence of 10 μm, 1 μm, 0.6 μm and 0.1 μm pore sizes had agglomerated flake-like morphologies (Fig. 1g). The morphologies of UHMWPE wear particles (Micro-wear-1.0–10 μm) isolated using a filter sequence of 10 μm and 1 μm pore sizes with an expected size range of 1.0–10 μm are shown in Fig. 1h. Large flake-like particles were observed, while a large number of nanometre-sized UHMWPE particles were also present on the filter (Fig. 1h).

**Fig. 2:**
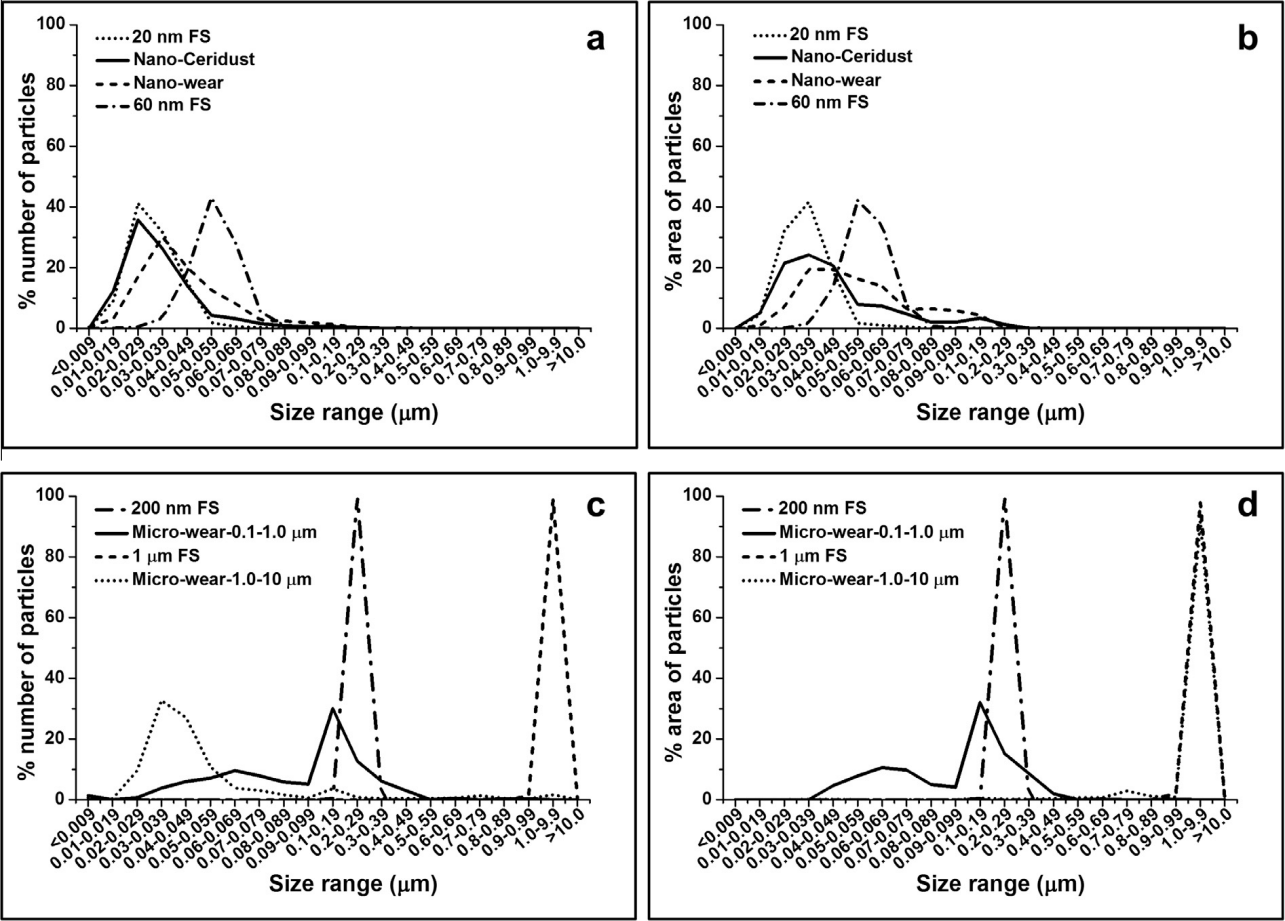
The percentage (a) number and (b) area distributions of 20 nm and 60 nm FS, compared to nanometre-sized Ceridust 3615® (Nano-Ceridust) and nanometre-sized UHMWPE wear particles (Nano-wear); The percentage (c) number and (d) area distributions of 200 nm and 1 μm FS, compared to micrometre-sized UHMWPE wear (Micro-wear-0.1–1.0 μm) and micrometre-sized UHMWPE wear particles (Micro-wear-1.0–10 μm).

The number and area distributions of nanometre-sized polymer particles as a function of size are shown in Fig. 2a and b. The nanometre-sized UHMWPE wear particles had a mode number in the 30–39 nm size range (Fig. 2a). For nanometre-sized Ceridust 3615®, the mode of the number of particles was in the 20–29 nm size range (Fig. 2a). The 20 nm and 40 nm FS showed narrower number and area distributions compared to those of fractionated nanosized UHMWPE wear particles and Ceridust particles (Fig. 2a and b). The mode of the number of 20 nm FS was in the size range of 20–29 nm (Fig. 2a). The mode of the number of 40 nm FS was in the size range of 50–59 nm (Fig. 2a). The majority of 40 nm FS particles were in the size range of >50 nm, while particles with a size range of <50 nm occupied the majority of the number of all the other three types of particles including 20 nm FS, nanometre-sized UHMWPE wear particles and nanometre-sized Ceridust 3615® particles. The 40 nm FS are therefore referred as 60 nm FS for the remainder of this paper.

The number and area distributions of micrometre-sized polymer particles as a function of size are shown in Fig. 2c and d. The 200 nm FS and 1 μm FS showed narrower distributions compared to fractionated UHMWPE wear particles. All of the 200 nm FS were within a size range of 0.2–0.29 μm and all of the 1.0 μm FS were within a size range of 1.0–2.0 μm. The mode of both the number and the area distributions of micrometre-sized UHMWPE wear particles (Micro-wear-0.1–1.0 μm) were in the size range of 0.1–0.19 μm. The micrometre-sized UHMWPE particles with an expected size range of 1.0–10 μm (Micro-wear-1.0–10 μm) had a mode number in the size range of 30–39 nm (Fig. 2c). Particles with a size range of 1.0–9.9 μm represented only 1.6% of the number of particles (Fig. 2c), however, these particles accounted for 91.8% of the area of particles (Fig. 2d).

### 3.2 Endotoxin levels of polymer particles

All the particle stocks including each size of FS, fractionated Ceridust 3615® particles and UHMWPE wear particles showed minimum endotoxin levels of <0.5 Endotoxin Units (EU) ml^−1^, which were below the accepted value of <5 Endotoxin Units (EU kg^−1^) according to the specification of the endotoxin tolerance limit for non-pyrogenic products for the pharmaceutical industry (FDA Regulatory Affairs, 1985).

### 3.3 Cellular responses to polymer particles in terms of osteolytic cytokine release

#### 3.3.1 Cell viability

Results obtained from the ATP Lite cell viability assay indicated that there was no adverse effect on cell viability within any of the treatment groups compared to the negative control (cells only), data not shown.

#### 3.3.2 Cellular responses to FluoSphere particles

**Fig. 3:**
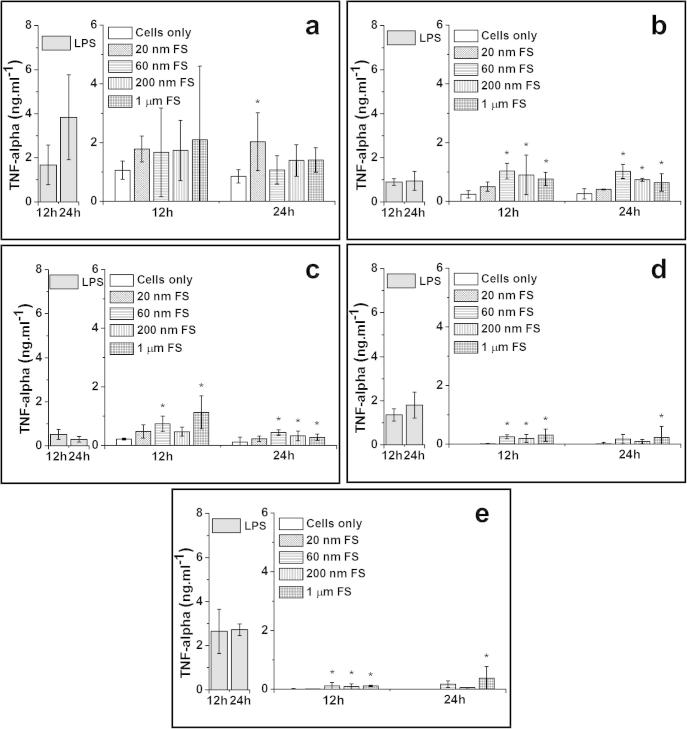
TNF-α released (mean ± 95% confidence intervals, *n* = 3) by PBMNCs from 5 donors after stimulation with 100 μm^3^ of 20 nm, 60 nm, 200 nm and 1.0 μm FS per cells over 12 h and 24 h: (a) Donor 1; (b) Donor 2; (c) Donor 3; (d) Donor 4 and (e) Donor 5. Positive control: 200 ng ml^−1^ of LPS; Negative control: Cells only. ^*^Indicates a statistically significant elevation of cytokines compared to the cells only control (ANOVA, *p* < 0.05).

**Fig. 4:**
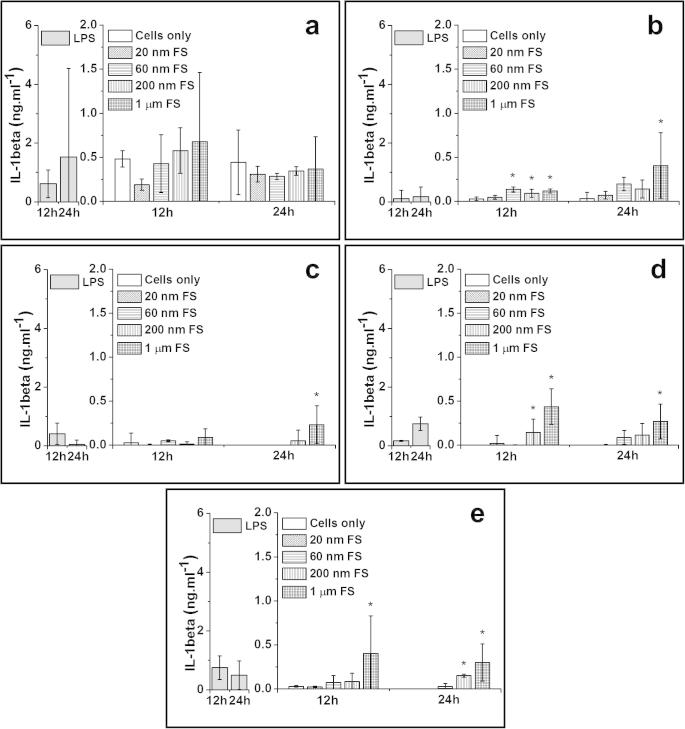
IL-1β released (mean ± 95% confidence intervals, *n* = 3) by PBMNCs from 5 donors after stimulation with 100 μm^3^ of 20 nm, 60 nm, 200 nm and 1.0 μm FS per cells over 12 h and 24 h: (a) Donor 1; (b) Donor 2; (c) Donor 3; (d) Donor 4 and (e) Donor 5. Positive control: 200 ng ml^−1^ of LPS; Negative control: Cells only. ^*^Indicates a statistically significant elevation of cytokines compared to the cells only control (ANOVA, *p* < 0.05).

**Fig. 5:**
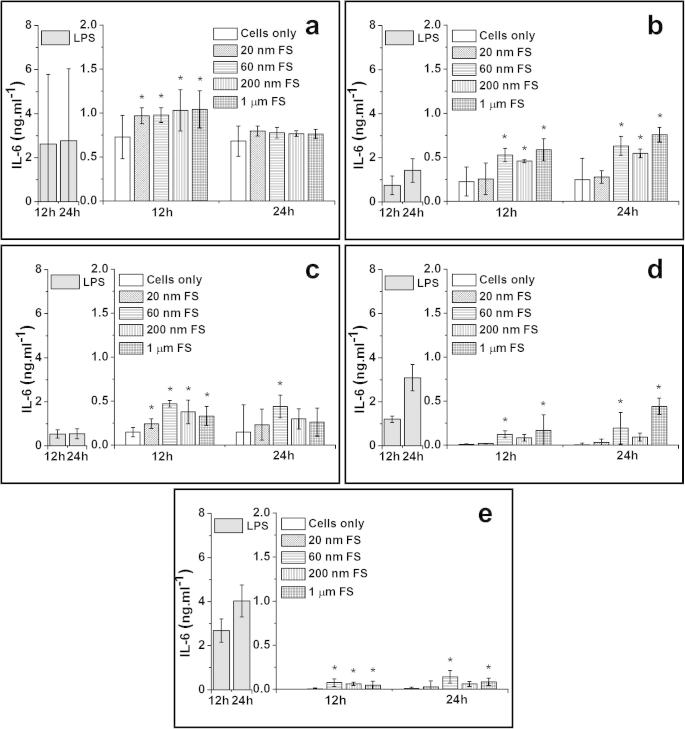
IL-6 released (mean ± 95% confidence intervals, *n* = 3) by PBMNCs from 5 donors after stimulation with 100 μm^3^ of 20 nm, 60 nm, 200 nm and 1.0 μm FS per cells over 12 h and 24 h: (a) Donor 1; (b) Donor 2; (c) Donor 3; (d) Donor 4 and (e) Donor 5. Positive control: 200 ng ml^−1^ of LPS; Negative control: Cells only. ^*^Indicates a statistically significant elevation of cytokines compared to the cells only control (ANOVA, *p* < 0.05).

**Fig. 6:**
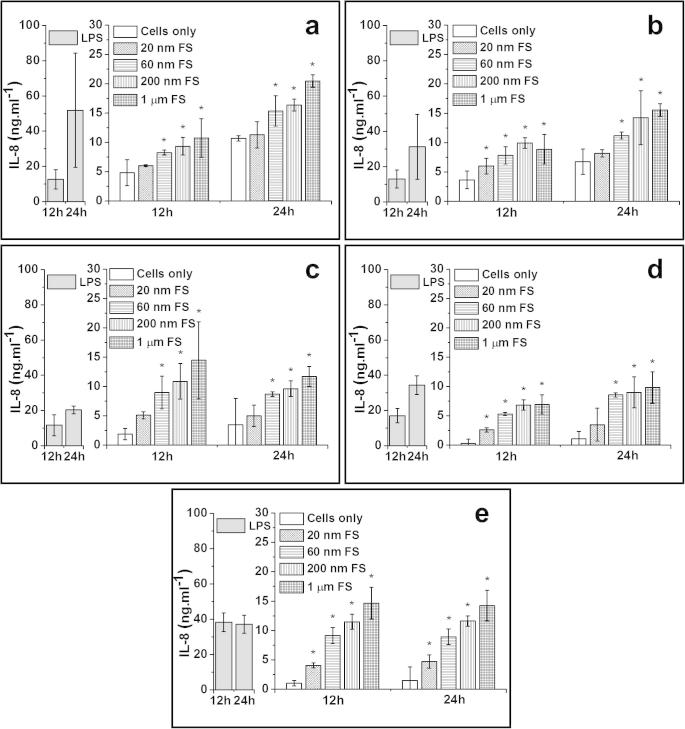
IL-8 released (mean ± 95% confidence intervals, *n* = 3) by PBMNCs from 5 donors after stimulation with 100 μm^3^ of 20 nm, 60 nm, 200 nm and 1.0 μm FS per cells over 12 h and 24 h: (a) Donor 1; (b) Donor 2; (c) Donor 3; (d) Donor 4 and (e) Donor 5. Positive control: 200 ng ml^−1^ of LPS; Negative control: Cells only. ^*^Indicates a statistically significant elevation of cytokines compared to the cells only control (ANOVA, *p* < 0.05).

PBMNCs isolated from five donors were cultured with 100 μm^3^ per cell of the 20 nm, 60 nm, 200 nm and 1.0 μm sized FluoSpheres (FS) for 12 and 24 h. The results for TNF-α, IL-1β, IL-6 and IL-8 release by the cells isolated from five donors are shown in Figs. 3–6, respectively. A summary of donors that were stimulated to release significant elevations of cytokines compared to cells only control is shown in Table 1.

##### 3.3.2.1 TNF-α release from particle-stimulated PBMNCs

The 1.0 μm sized FS stimulated cells from 4/5 donors to produce significantly higher levels of TNF-α after 12 and 24 h compared to the cells only negative control. The 200 nm FS caused significantly elevated production of TNF-α released by cells from 3/5 donors at the 12 h time point and cells from 2/5 donors at the 24 h time point. The 60 nm FS caused significant elevation of TNF-α release by cells from 4/5 donors at 12 h and cells from 2/5 donors at 24 h. The 20 nm FS only stimulated cells from 1/5 donor to produce significantly higher levels of TNF-α at the 24 h time point compared to the cells only control.

##### 3.3.2.2 IL-1β release from particle-stimulated PBMNCs

The 1.0 μm sized FS stimulated significantly higher amounts of IL-1β released by cells from 3/5 donors at the 12 h time point and cells from 4/5 donors at the 24 h time point, compared to the cells only control. The 200 nm FS caused significant elevation of IL-1β production by cells from 2/5 donors at 12 h and 1/5 donor at 24 h. The 60 nm FS stimulated cells from 1/5 donor to release higher amounts of IL-1β at the 12 h time point, compared to the control. For the 20 nm FS, there was no significant production of IL-1β by cells from any of the five donors at any time point.

##### 3.3.2.3 IL-6 release from particle-stimulated PBMNCs

For 1.0 μm sized FS, there was significant production of IL-6 by cells from all donors after 12 h and cells from 3/5 donors after 24 h. The 200 nm FS caused significant elevation of IL-6 release by cells from 4/5 donors at 12 h and 1/5 donor at 24 h time point. The 60 nm FS caused higher amounts of IL-6 released by cells from all donors at the 12 h time point, and 4/5 donors at the 24 h time point, compared to the control. The 20 nm FS only stimulated significant elevation of IL-6 released by cells from 2/5 donors at 12 h time point. The 20 nm FS did not cause any significant production of IL-6 by cells from any donors after 24 h.

##### 3.3.2.4 IL-8 release from particle-stimulated PBMNCs

The 1.0 μm sized FS caused significant elevation of IL-8 production by cells from all donors at both of 12 h and 24 h time points. Similar trends were observed for cells treated with 200 nm FS and 60 nm FS which caused significant higher amounts of IL-8 released by cells from all donors after 12 h and 24 h, compared to the control. For the 20 nm FS, significant production of IL-8 was only observed in cells from 3/5 donors at 12 h and cells from 1/5 donor at 24 h.

#### 3.3.3 Comparison of osteolytic cytokine release stimulated by nanometre-sized polymer particles and micrometre-sized polymer particles

**Fig. 7:**
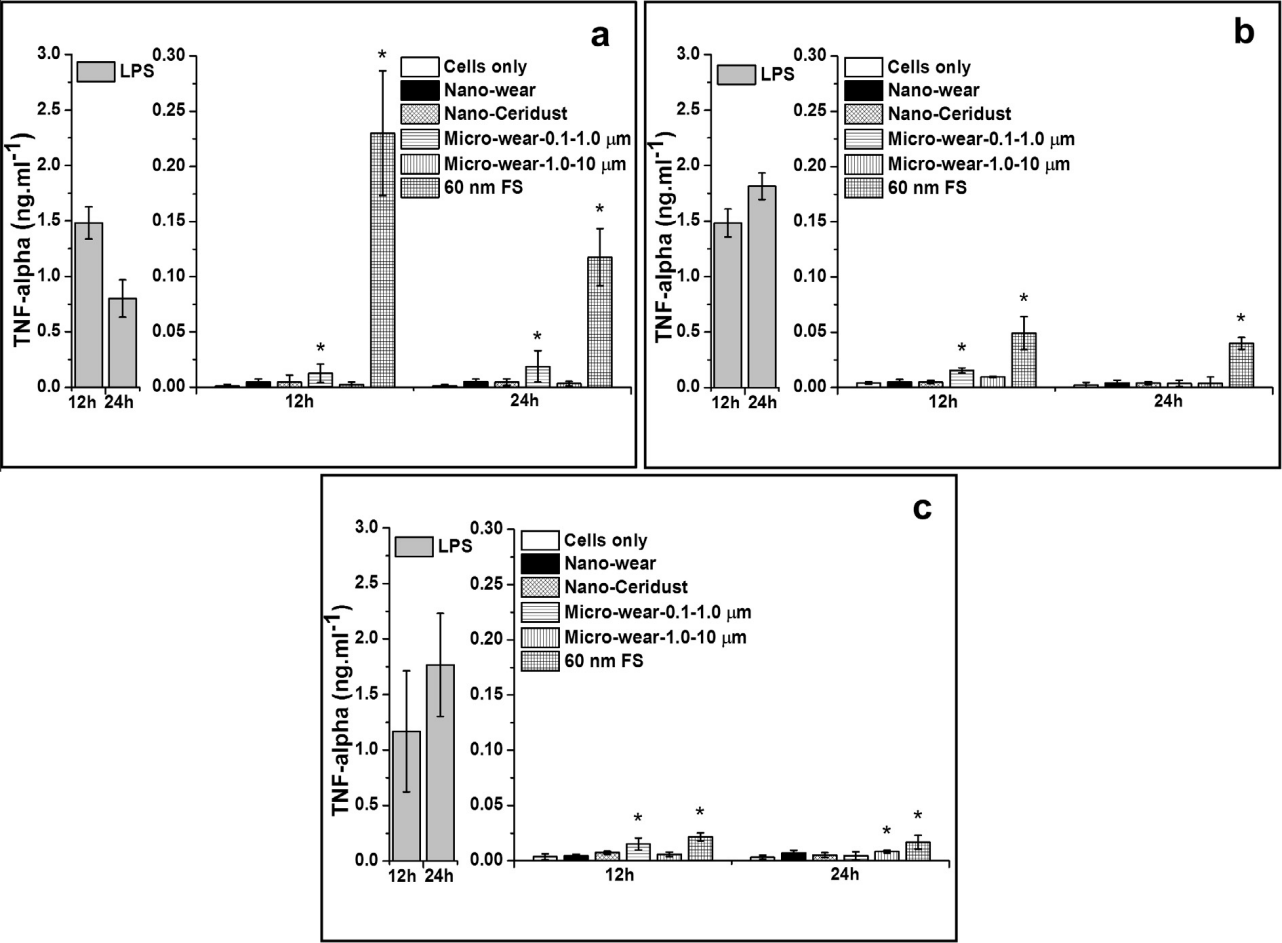
TNF-α released (mean ± 95% confidence intervals, *n* = 3) by PBMNCs from 3 donors after stimulation with nanometre-sized and micrometre-sized polymer particles at a dose of 100 μm^3^ particles per cell over 12 and 24 h: (a) Donor 1; (b) Donor 2; (c) Donor 3. ^*^Indicates a statistically significant elevation of cytokines compared to the cells only negative control (ANOVA, *p* < 0.05).

**Fig. 8:**
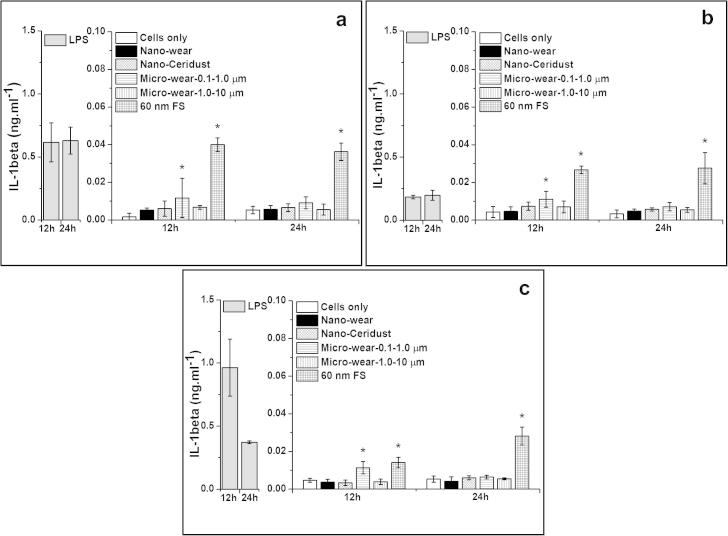
IL-1β released (mean ± 95% confidence intervals, *n* = 3) by PBMNCs from 3 donors after stimulation with nanometre-sized and micrometre-sized polymer particles at a dose of 100 μm^3^ particles per cell over 12 and 24 h: (a) Donor 1; (b) Donor 2; (c) Donor 3. ^*^Indicates a statistically significant elevation of cytokines compared to the cells only negative control (ANOVA, *p* < 0.05).

**Fig. 9:**
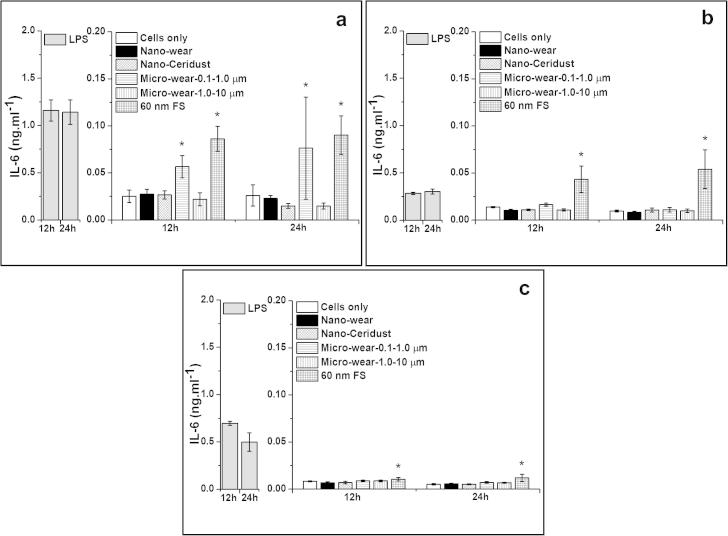
IL-6 released (mean ± 95% confidence intervals, *n* = 3) by PBMNCs from 3 donors after stimulation with nanometre-sized and micrometre-sized polymer particles at a dose of 100 μm^3^ particles per cell over 12 and 24 h: (a) Donor 1; (b) Donor 2; (c) Donor 3. ^*^Indicates a statistically significant elevation of cytokines compared to the cells only negative control (ANOVA, *p* < 0.05).

**Fig. 10:**
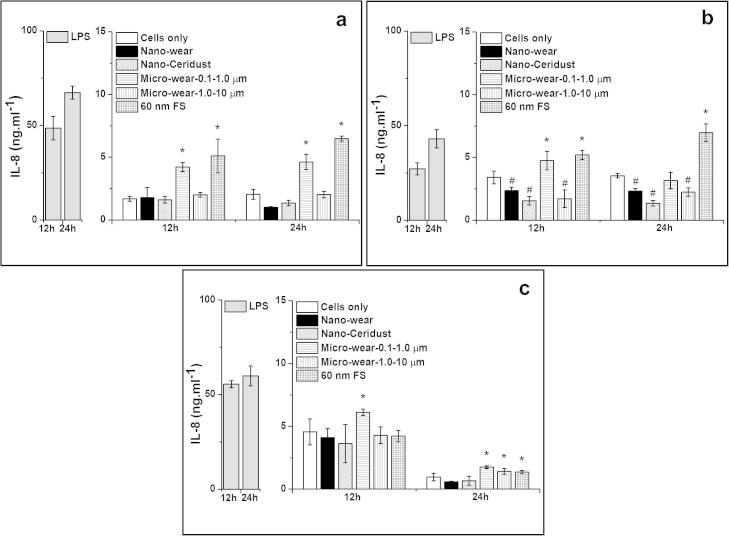
IL-8 released (mean ± 95% confidence intervals, *n* = 3) by PBMNCs from 3 donors after stimulation with nanometre-sized and micrometre-sized polymer particles at a dose of 100 μm^3^ particles per cell over 12 and 24 h: (a) Donor 1; (b) Donor 2; (c) Donor 3. ^*^Indicates a statistically significant elevation of cytokines compared to the cells only negative control. ^#^Indicates a statistically significant lower level of cytokines compared to the cells only control (ANOVA, *p* < 0.05).

PBMNCs isolated from three donors were cultured with 100 μm^3^ per cell of nanometre-sized polymer particles and micrometre-sized polymer particles for 12 and 24 h. The results for TNF-α, IL-1β, IL-6 and IL-8 release by the cells isolated from three donors are shown in Figs. 7–10, respectively. A summary of donors that were stimulated to release significant elevations of cytokines compared to cells only control is shown in Table 2.

##### 3.3.3.1 TNF-α release from particle-stimulated PBMNCs

The 60 nm FS caused significantly higher levels of TNF-α release from all three donors after incubation for 12 h and 24 h compared to the cells only negative control. Micrometre-sized UHMWPE wear particles (Micro-wear-0.1–1.0 μm) stimulated significant elevation of TNF-α release by cells isolated from 3/3 donors at the 12 h time point and cells from 1/3 donors at the 24 h time point. Significantly elevated levels of TNF-α were released by cells from 1/3 donors in response to the micrometre-sized UHMWPE wear particles (Micro-wear-1.0–10 μm) at the 24 h time point. The nanometre-sized UHMWPE wear particles and nanometre-sized Ceridust 3615® did not stimulate cells from any of the three donors to produce elevated levels of TNF-α at any time point, compared to the cells only negative control.

##### 3.3.3.2 IL-1β release from particle-stimulated PBMNCs

The 60 nm FS caused significantly elevated levels of IL-1β release by cells from all three donors at both time points tested (12 h and 24 h). Cells from 3/3 donors treated with micrometre-sized UHMWPE wear particles (Micro-wear-0.1–1.0 μm) secreted significantly elevated concentrations of IL-1β after 12 h, but no significantly increased release of IL-1β was observed at 24 h. The nanometre-sized UHMWPE wear particles, nanometre-sized Ceridust 3615® and micrometre-sized UHMWPE wear particles (Micro-wear-1.0–10 μm) failed to stimulate significantly elevated levels of IL-1β release from cells from any of the three donors at either time point compared to the negative control.

##### 3.3.3.3 IL-6 release from particle-stimulated PBMNCs

The 60 nm FS stimulated significantly higher amounts of IL-6 release by cells from 3/3 donors at both time points tested (12 h and 24 h), compared to the cells only control. The micrometre-sized UHMWPE wear particles (Micro-wear-0.1–1.0 μm) caused significantly elevated IL-6 release by cells from 1/3 donor after 12 h and 24 h. Neither micrometre-sized UHMWPE wear particles (Micro-wear-1.0–10 μm), nanometre-sized UHMWPE wear particles or nanometre-sized Ceridust 3615® caused any significant elevation of IL-6 at 12 h or 24 h, compared to the cells only negative control.

##### 3.3.3.4 IL-8 release from particle-stimulated PBMNCs

There was abundant IL-8 secreted from cells without particle stimulation (negative control ranges from 0.96–4.57 ng ml^−1^). The 60 nm FS stimulated significantly higher levels of IL-8 release by cells from 2/3 donors at 12 h and 3/3 donors at 24 h, compared to the negative control. The micrometre-sized UHMWPE wear particles (Micro-wear-0.1–1.0 μm) provoked significantly high amounts of IL-8 release at the 12 time point (3/3 donors) and at the 24 time point (2/3 donors). The micrometre-sized UHMWPE wear particles (Micro-wear-1.0–10 μm) stimulated a significantly high level of IL-8 release by cells from 1/3 donors at the 24 h time point. No significant increase in the production of IL-8 by cells from all three donors was observed for nanometre-sized UHMWPE wear particles and nanometre-sized Ceridust 3615® at both time points.

### 3.4 Visualisation of nanometre-sized and micrometre-sized UHMWPE wear particles uptake by PBMNCs

**Fig. 11:**
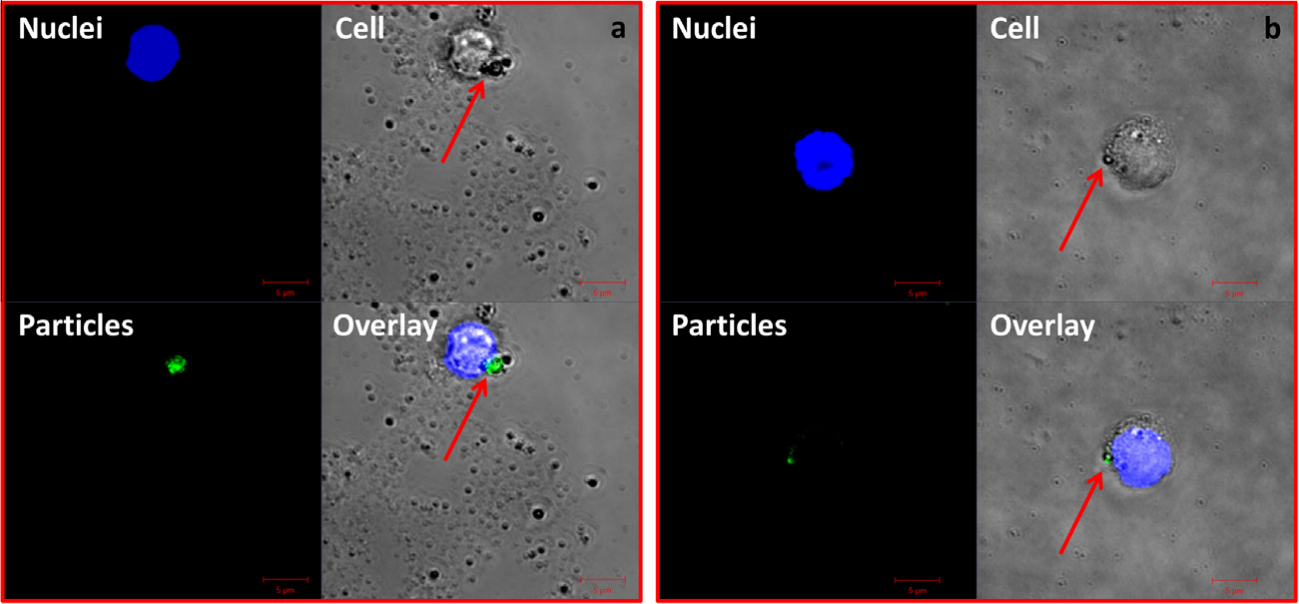
Visualisation of (a) nanometre-sized UHMWPE wear particles and (b) 0.1–1.0 micrometre-sized UHMWPE wear particles internalised by PBMNCs cultured on agarose gel. Confocal images of single sections through cells are shown. Blue signals represent nuclei stained with Hoechst 33342 and green signals represent fluorescently labelled nanometre-sized and micrometre-sized UHMWPE particles. Arrows indicate particle aggregates. Size bar: 5 μm.

Confocal images (Fig. 11) showed cells with blue nuclei and internalised green fluorescently labelled nanometre-sized and micrometre-sized UHMWPE wear particles. The confocal images were taken in the midsection of the cells to ensure that the fluorescence signal observed was from particles that had been endocytosed by PBMNCs. For cells plus nanometre-sized particles, the bright punctate fluorescence particles were observed as spots accumulated in the perinuclear region, which indicated the agglomerated nanometre-sized UHMWPE wear particles had been engulfed by cells (Fig. 11a). The particles presented as black dots within cells under bright field illumination, as shown by the red arrows. The green spots indicated the localisation of micrometre-sized UHMWPE wear particles within the cells, which were also localised around the periphery of the nuclei (Fig. 11b).

## 4 Discussion

It has been shown that nanometre-sized metal particles generated by metal-on-metal prostheses have the potential to play a role in adverse tissue reactions such as tissue necrosis and the development of pseudotumors [11,29,30]. Compared to metal particles, UHMWPE is a chemically inert material and phagocytic cells are not able to degrade the particles once they have been internalised, in spite of the low pH of the intracellular compartment. Even though nanometre-sized UHMWPE wear particles occupy a relatively small percentage of the total wear volume generated by conventional THR, they are produced in large numbers. Due to their small size, numerous nanometre-sized UHMWPE wear particles may be disseminated throughout the body and UHMWPE wear particles have previously been found in patient lymph nodes, spleen and liver [31–33]. There are no published reports on the biological response to nanometre-sized UHMWPE wear particles and it is unclear whether nanometre-sized UHMWPE may contribute to osteolysis in THR. This may be due to the difficulty in obtaining sufficient amounts of clinically relevant nanometre-sized UHMWPE wear particles for study.

Recently, Pal et al. [34] reported that nanoscale UHMWPE wear particles caused an inflammatory response in terms of IL-6 and IL-1β release from dendritic cells (DCs). However, the nanoscale UHMWPE wear particles used in that study had a size range of 0.05–0.2 μm, which are not strictly nanometre-sized particles (size range of <0.1 μm). The proportion of particles within a size range of 0.1–0.2 μm which has previously been shown to be the most biologically active [16,24] might mask the cellular response to particles within the true nanometre-size range. Obviously, the results from the study by Pal et al. [34] did not convincingly answer the questions regarding the biological response to nanometre-sized UHMWPE wear particles. In the present study, the aim was to investigate only nanometre-sized UHMWPE wear particles i.e. those smaller than 100 nm, in isolation and to determine their contribution to osteolysis.

In the present study, a large volume of sterile clinically-relevant nanometre-sized UHMWPE wear particles with a mode size of 30–40 nm was successfully isolated from lubricants generated in a multidirectional six station pin-on-plate simulator, which was operated in a non-sterile environment. The nanometre-sized UHMWPE particles had a granular morphology and tended to agglomerate, which was consistent with particles isolated from periprosthetic tissues of patients with failed THR [19,20]. The majority of nanometre-sized particles isolated in the present study were less than 50 nm in size, which was similar to particle distributions reported in a study on particle analysis from retrieval tissue from THR patients [20]. The flake like shape of micrometre-sized UHMWPE particles (Micro-wear-0.1–1.0 μm) isolated from the wear test lubricants were also similar to particles isolated from the retrieved tissues of patients at revision hip surgery [35]. This indicated that the six station pin-on-plate simulation was an efficient and reliable way of generating endotoxin free clinically-relevant UHMWPE wear particles with different size ranges for use in *in vitro* biological response studies.

The fractionated micrometre-sized UHMWPE wear particles (Micro-wear-0.1–1.0 μm) had more than 50% of particles within a size range of 0.1–1.0 μm and the micrometre-sized UHMWPE wear particles (Micro-wear-1.0–10 μm) comprised 91.8% of the area of particles within the size range of 1.0–10 μm. These two types of particles represented particles within the size range of 0.1–10 μm which had previously been reported to be the most biologically active [16,27]. This enabled a direct comparison of levels of osteolytic cytokine release from cells stimulated by nanometre-sized and micrometre-sized UHMWPE particles. In addition to size and volume, other factors such as composition, surface area, surface texture and morphology of wear debris are also thought to influence this macrophage-mediated osteolysis process [36,37]. Therefore, the cellular response to model particles including FluoSpheres® (FS) and Ceridust 3615® which had different particle composition and morphologies to UHMWPE wear particles was also investigated in this study.

All size ranges of FluoSphere particles showed similar regular round morphologies in SEM images, therefore, their cellular response was mainly determined by size distribution. The cellular response results indicated that 1 μm FS (mode size 1.0–2.0 μm) were the most biologically active particles, while 20 nm FS (mode size 20–30 nm) were the least biologically active, which was consistent with the conclusion from our previous study, which showed that UHMWPE particles within 0.1–10 μm size range are the most biologically active [27]. For Fluosphere particles, the 1 μm FS showed higher biological activity than 200 nm FS, however, the UHMWPE wear particles did not follow this trend. The 0.1–1.0 micrometre-sized UHMWPE wear particles stimulated significantly higher levels of TNF-α, IL-1β and IL-8 release by cells from 3/3 donors and IL-6 by cells from 1/3 donors at 12 h. In addition, these particles stimulated significantly elevated TNF-α and IL-6 release for cells from 1/3 donors, and IL-8 release by cells from 2/3 donors at 24 h. However, the 1.0–10 micrometre-sized UHMWPE wear particles only stimulated cells isolated from 1/3 donors to produce significantly higher levels of TNF-α and IL-8 at 24 h. Previous observations have shown that the proportion of particles which are within the most biologically active size range (0.1–1.0 μm) is a critical determinant in the biological activity of the UHMWPE particles [14,22]. Therefore, the lower biological activity of the 1.0–10 micrometre-sized UHMWPE wear may be explained by the fact that the 1.0–10 micrometre-sized UHMWPE wear particles had a lower proportion of particles that were in the biologically active size range (0.1–1.0 μm) compared to the 0.1–1.0 micrometre-sized UHMWPE wear particles.

Neither nanometre-sized Ceridust 3615® with a mode size of 20–30 nm or nanometre-sized UHMWPE wear particles with a mode size of 30–40 nm caused a significant increase in the production of osteolytic cytokines by cells from 3/3 donors at any time point, compared to the cells only control. This was consisted with the results observed from 20 nm FS which demonstrated the lowest cellular activation among all size range of FS. All these results indicated that there was a lower size limit for cellular activation regardless of the composition and morphology of particles.

However, there was difference among the cellular response to a similar size range of particle with different composition and shapes. Although the 20 nm FS had a similar distribution (as a function of size) compared to the nanometre-sized Ceridust, the 20 nm FS had higher biological activity. Compared to nanometre-sized Ceridust 3615®, the 20 nm FS were manufactured from a different material, polystyrene, they also had a more regular shape and narrower size distribution, which might play a role in the different biological activity of these two types of particles. For micrometre-size particles, the 200 nm FS and 1.0 μm FS showed higher biological activity than 0.1–1.0 micrometre-sized and 1.0–10 micrometre-sized UHMWPE wear particles, which also support the conclusion that composition and shape of particles might play an important role in determining the cellular response.

The levels of cytokine released by PBMNCs stimulated by micrometre-sized UHMWPE wear particles (Micro-wear-0.1–1.0 μm) were much lower than those measured when cells were stimulated by 60 nm FS at the same dose. These two types of particles had different size distributions, different compositions and shapes. The 0.1–1.0 micrometre-sized UHMWPE wear particles had high proportions of the number and area of particles within the critical size (0.1–1.0 μm), but they had a much broader size distribution of particles compared to the 60 nm FS. The large number and volume of particles outside of the critical size range might have a significant effect on cytokine release by cells stimulated with the 0.1–1.0 micrometre-sized UHMWPE wear particles. Compared to fractionated UHMWPE wear particles, the surface of the FS was much rounder and smoother in appearance, which might also have contributed to higher cellular response of 60 nm FS.

Overall, the results indicated that the size, composition and morphology of the particles all played an important role in the process of macrophage activation and the subsequent cytokine release.

The data presented also add to the evidence of the heterogeneity of human individuals in terms of their biological response to both model particles and UHMWPE wear particles. The concentration of each osteolytic cytokine released showed marked variation between the donors in the present study, which was in agreement with previous studies [16,24], where up to 15-fold differences in the concentration of cytokine was produced in response to the same dose of UHMWPE particles. This variation has been postulated to be due to cytokine promoter gene polymorphisms of different individuals [38]. In addition, in the present study, lower levels of IL-8 release were observed in cells from 1/3 donors in response to nanometre-sized UHMWPE wear particles, nanometre-sized Ceridust and micrometre-sized UHMWPE wear particles (Micro-wear-1.0–10 μm), which are also postulated to be due to donor specific responses.

The concentration of agarose gels used in this study was 0.3% (w/v) which has been shown to have a peak distribution of pore diameters of 8 μm (ranging from 2 μm to 13 μm) [39]. Monocytes are approximately 12–20 μm in diameter, therefore, the 0.3% (w/v) gel was not only able to provide a solid support for the particles but also offered an environment for the free migration of human monocytes within the gel. The confocal images show that both nanometre-sized and micrometre-sized UHMWPE wear particles were endocytosed by PBMNCs, which provides the evidence that PBMNCs cultured using the agarose gel method were able to take up nanometre-sized UHMWPE particles. No significant differences were observed in the number of nanometre-sized and micrometre-sized UHMWPE wear particles taken up by cells, which indicated that the gel did not play a role in determining the cellular response to UHMWPE wear particles. Therefore, the gel-particle-challenge method was feasible to investigate the biological activity of UHMWPE wear particles. In order to determine why the engulfed nanometre-sized UHMWPE particles did not cause an inflammatory response, further investigations are being carried out to determine the relationship between the cellular uptake mechanism and cellular response to different sizes of UHMWPE wear particles.

## 5 Conclusions

In this study sterile clinically-relevant nanometre-sized UHMWPE wear particles which had a size range of <50 nm, were successfully generated and isolated. The results showed for the first time that nanometre-sized UHMWPE wear particles with a size range of <50 nm did not stimulate proinflammatory cytokine release including TNF-α, IL-1β, IL-6 and IL-8, from human peripheral blood mononuclear cells (PBMNCs). This conclusion has answered long-term clinical concerns about the biological response to the large number of nanometre-sized UHMWPE wear particles which are generated from highly crosslinked UHMWPE acetabular cups in total hip replacements. In addition, this study indicated that size, composition and morphology of particles played an important role in the particle-induced osteolysis process. Further investigation to determine the cellular uptake mechanism of clinically-relevant nanometre-sized UHMWPE wear particles compared to micrometre-sized UHMWPE wear particles is underway with the aim of revealing the relationship between the cellular uptake mechanism and cellular response to different sizes of UHMWPE wear particles.

## Figures and Tables

**Table 1:** Cytokine release from 5 donors when stimulated with 20 nm, 60 nm, 200 nm and 1.0 μm FS particles at a concentration of 100 μm^3^ particles per cell over 12 and 24 h.

Particles	Cytokine release							
	12 h				24 h			
	TNF-α	IL-1β	IL-6	IL-8	TNF-α	IL-1β	IL-6	IL-8
20 nm FS	N	N	D1 D3	D2 D4 D5	D1	N	N	D5
60 nm FS	D2 D3 D4 D5	D2	D1 D2 D3 D4 D5	D1 D2 D3 D4 D5	D2 D3	N	D2 D3 D4 D5	D1 D2 D3 D4 D5
200 nm FS	D2 D4 D5	D2 D4	D1 D2 D3 D5	D1 D2 D3 D4 D5	D2 D3	D5	D2	D1 D2 D3 D4 D5
1.0 μm FS	D2 D3 D4 D5	D2 D4 D5	D1 D2 D3 D4 D5	D1 D2 D3 D4 D5	D2 D3 D4 D5	D2 D3 D4 D5	D2 D4 D5	D1 D2 D3 D4 D5

*Notes:* The symbols represent whether there was significant cytokine release. Where *N* = no significant cytokine release and D1, D2, D3, D4, D5 depict significant cytokine release: D1 = Donor 1; D2 = Donor 2; D3 = Donor 3; D4 = Donor 4; D5 = Donor 5.

**Table 2:** Cytokine release from 3 donors when stimulated with nanometre-sized and micrometre-sized polymer particles at a concentration of 100 μm^3^ particles per cell over 12 and 24 h.

Particles	Cytokine release							
	12 hours				24 hours			
	TNF-α	IL-1β	IL-6	IL-8	TNF-α	IL-1β	IL-6	IL-8
Nano-wear	N	N	N	N	N	N	N	N
Nano-Ceridust	N	N	N	N	N	N	N	N
Micro-wear-0.1–1.0 μm	D1 D2 D3	D1 D2 D3	D1	D1 D2 D3	D1	N	D1	D1 D3
Micro-wear- 1.0–10 μm	N	N	N	N	D3	N	N	D3
60 nm FS	D1 D2 D3	D1 D2 D3	D1 D2 D3	D1 D2	D1 D2 D3	D1 D2 D3	D1 D2 D3	D1 D2 D3

*Notes:* The symbols represent whether there was significant cytokine release. Where N = no significant cytokine release and D1, D2, D3 depict significant cytokine release: D1 = Donor 1; D2 = Donor 2; D3 = Donor 3.

## Appendix A Figures with essential colour discrimination

Certain figures in this article, particularly Fig. 11, are difficult to interpret in black and white. The full colour images can be found in the on-line version, at http://dx.doi.org/10.1016/j.actbio.2015.05.016.
